# Valorisation of Prosciutto di Parma PDO by-products for the development of protein-rich snacks

**DOI:** 10.3389/fnut.2026.1894965

**Published:** 2026-09-04

**Authors:** Filippo Rossi, Martina Cassarino, Paolo Valoti, Giuliano Dall'Olio, Edoardo Fornari, Gianluca Giuberti, Gabriele Rocchetti, Ilaria Zampieri, Giulia Leni, Margherita Dall'Asta

**Affiliations:** 1 Dipartimento di Scienze Animali, della Nutrizione e degli Alimenti, Istituto di Scienze degli Alimenti e della Nutrizione, Facoltà di Scienze Agrarie, Alimentari e Ambientali, Università Cattolica del Sacro Cuore, Piacenza, Italy; 2 Consiglio per la Ricerca in Agricoltura e l'Analisi dell'Economia Agraria, Centro di Ricerca Cerealicoltura e Colture Industriali, Bergamo, Italy; 3 Dipartimento di Scienze e Tecnologie Alimentari per una filiera agro-alimentare Sostenibile – DiSTAS, Facoltà di Scienze Agrarie, Alimentari e Ambientali, Università Cattolica del Sacro Cuore, Piacenza, Italy; 4 Dipartimento di Economia Agro-Alimentare, Facoltà di Scienze Agrarie, Alimentari e Ambientali, Università Cattolica del Sacro Cuore, Piacenza, Italy; 5 CriBeNS - Centro di ricerca in Biochimica e Nutrizione dello Sport, Università Cattolica del Sacro Cuore, Rome, Italy

**Keywords:** circularity, cured pork meat, polyphenols, protein quality, starch

## Abstract

**Introduction:**

There is a growing demand for high-protein products from people who play sports, want to lose weight, or need to gain muscle mass. Among the most useful protein sources are meat-processed products, which, however, have a high environmental impact. The aim of the work was to create a snack rich in proteins, which allows the valorization of the residues of the slicing of raw Parma Ham a typical Italian product labeled as POD (Protected Designation of Origin), which are, currently, sold at low prices for the production of animal feed or stuffed pasta.

**Methods:**

Six different types of bars were developed, they were produced with raw ham residues (35%), pork, water, yellow corn with a high amylose content (AE), or rich in polyphenols (red or blue), pork gelatine/Psyllium fiber, flavorings. The following items were determined on the bars: centesimal composition, *in vitro* carbohydrate digestion, *in vitro* protein digestibility, polyphenols content. Statistical analysis was performed with Tukey test (*p* < 0.05).

**Results:**

The composition of the bars was in this range (as fed): kcal 279 – 313; proteins 26.7%−38.1%; lipids 10.2%−12.2%; carbohydrates 14.9%−18.4%. FAO protein score (ProteinScore) on the digested fraction was greater with red corn (79 Leu) than with blue (64 Trp) and AE (64 Trp).

**Conclusions:**

All bars can use the claim “Rich in Protein” as defined by EFSA, the prototype AE Psyllium 5% showed the best resistant starch levels and protein score.

## 1 Introduction

Italy is one of the leading countries of PDO and PGI products, in particular processed meats. In this context, pre-packed sliced cured pork meat is an economically significant market, considering that according to real sales data provided by Circana Unify+ (1), it represents the first product category out of 469 Fast Moving Consumer Good (FMCG) markets sold in the Modern Grocery Distribution (MGD) channel in terms of value sales, as its turnover reached 2.8 Euro billion in 2024 with a +4% CAGR in the 5-year timespan 2020–2024. Within this industry, the raw ham segment is particularly important, since it is the second biggest after that of cooked ham, with a turnover of 499 Euro million in 2024 (+3% CAGR in the 2020–2024 timespan), corresponding to a total sales volume of 17,350 tons and an average selling price of 28,80 Euro/Kg, over 10 €/kg higher than other types of cured pork meats. In the raw ham segment, the main player is Prosciutto di Parma PDO. In 2022, the turnover of the Prosciutto di Parma Consortium was 800 million euros (with a production of 7,850,000 branded hams and an export value of 36%).

In recent years, the demand of ready-to-eat sliced products has increased, leading to an intensification of slicing and packaging operations. As a result, losses associated with the production of these products are estimated to account for approximately 10% of the total volume of the original product, with a significant impact from both an environmental and economic point of view. In this context, adopting a circular economy approach becomes essential, both to decrease food waste and to valorize these by-products, which are rich in proteins and micronutrients.

In line with these needs, the European Union (EU) is actively promoting strategies aimed at reducing food loss and waste (FLW) and identifying innovative end-uses for food by-products (2). This is particularly relevant for the meat industry, which is highly vulnerable to FLW due to its complex supply chain (3). The valorization of slicing by-products in the processed meat sector can contribute to improving economic returns, reducing environmental burdens, and enhancing the resilience of the supply chain, in line with circular principles (4).

From a nutritional perspective, this approach is especially promising, as meat-derived-by products are naturally rich in high-quality proteins and essential amino acids, including branched chain amino acids (BCAAs) (5). Protein quality depends on amino acid composition and digestibility, with the content and bioavailability of essential amino acids being key determinants of nutritional value (6). BCAAs, especially leucine, play a central role in stimulating muscle protein synthesis, and their anabolic effect is enhanced when adequate energy sources, such as carbohydrates, are available.

This anabolic role of BCAAs is maximized when carbohydrate sources, which provide the energy needed for protein synthesis, are also present. Consequently, combining carbohydrates with the cured meat processing residues could maximize the anabolic role of BCAAs (7).

Similar to protein, carbohydrate quality is also important from a nutritional perspective. Studies show that pigmented maize presents better sensory and nutritional characteristics than other varieties. Amylose-extender corn, in particular, is well known for its lower starch digestibility and, consequently, a lower glycemic index compared to other cultivars (8). Pigmented corn are rich in bioactive compounds as anthocyanins, flavonols, and carotenoids, which have increased interest among consumers in recent years due to their beneficial effects on reducing the risk of cancer, cardiovascular disease, and diabetes (9, 10).

Therefore, this project aims to develop a circularity-based approach for the reuse and valorization of meat by-products derived from one of the two most widely produced PDO and PGI products at the national level in Italy, through the development of novel high-protein snacks with enhanced nutritional profile.

## 2 Material and methods

### 2.1 Ingredients and sample preparation

Six different types of high protein bars have been developed. All bars were produced using seasoned raw ham residues (35%), fresh raw pork meat, and a source of starch such as yellow corn with a high amylose content (AE), or red pigmented corn (cv Morado, RED), or blue pigmented corn (cv blu/azul, BLU) (Research Center for Cereal and Industrial Crop, Italian Ministry of Agriculture, Bergamo, Italy). The three types of corn kernels were ground with a 1 mm screen using a Pulverissette 14 mill (Fritsch, Idar-Oberstein, Germany).

Two different gelatinizing agents were used: a pork gelatine (GEL) (Salumificio Fontana, Sala Baganza, Italy) and a plant-based alternative (psyllium fiber (PSY) (Psyllogel^®^, Giuliani, Milan, Italy). Detailed list of ingredients used for bars are reported in Table 1.

**Table 1:** List of snack bars ingredients.

Ingredients (g/100 g)	Type of bars					
	RED-GEL 5	BLU-GEL 5	AE-GEL 5	AE-PSY 5	AE-GEL 10	AE-PSY 10
Ham slicing residue	35	35	35	35	35	35
Raw minced pork meat	15	15	15	15	10	10
Water	24	24	24	24	24	24
Corn, yellow AE	–	–	20	20	20	20
Corn, blue	–	20	–	–	–	–
Corn, red	20	–	–	–	–	–
Jelly	5	5	5	–	10	–
Psyllium fiber	–	–	–	5	–	10
Lemon juice	1	1	1	1	1	1

The procedure for the manufacture of the bars was as follows: (a) the residues from slicing the raw ham and the raw pork meat were ground separately (with a 3 mm grid with a meat grinder ProPower, Bosch, Germany) and then mixed together in mixer (Pastamatic, De Longhi, Treviso, Italy); (b) after adding the gelatin, corn was added; (c) finally, water and lemon juice were added and the mixture was mixed for 1 min; (d) bars were obtained from the mixture, which were cooked in the oven at 110 °C for 2.5 h (oven FD53, WTC Binder, Tuttlingen, Germany).

The following six bars were produced:

- RED-GEL 5, manufactured with red corn and 5% of pork gelatine;
- BLU-GEL 5, manufactured with blu corn and 5% of pork gelatine;
- AE-GEL 5, manufactured with yellow, amylose extender corn and 5% of pork gelatine;
- AE-PSY 5, manufactured with yellow, amylose extender corn and 5% of psyllium fiber;
- AE-GEL 10, manufactured with yellow, amylose extender corn and 10 % of pork gelatine;
- AE-PSY 10 manufactured with yellow, amylose extender corn and 10 % of psyllium fiber;

### 2.2 Chemical composition

The bars were analyzed for their protein, fat, carbohydrate, and fiber content using the methods proposed by AOAC (11). Energy content (kcal) has been calculated using the energy yields for different nutrients (carbohydrates, lipids, protein, and fiber) established by the European Union (12).

Amino acids content of bars was determined by HPLC using the following method (13). For each product 0.5 g of sample were hydrolysed with 6 ml of HCl 6 N at 110 °C for 23 h, then the internal standard (7.5 ml of Norleucine 5 mm in water) was added. For cysteine and methionine, performic acid oxidation followed by acid hydrolysis was used to determine them as cysteic acid and methionine sulphone. In this case, an amount of 0.5 g of sample was mixed with 2 ml of performic acid freshly prepared (by combining 9 volumes of formic acid with 1 volume of hydrogen peroxide). Samples were kept in an ice bath for 16 h at 0°C and then 0.3 ml of hydrobromidric acid was added and the bromine formed was removed under nitrogen flow. The hydrolyzed samples were analyzed after derivatization with an AccQ Tag reagent (Waters Co., Milford, USA), as suggested by the supplier. Aminoacids were then analyzed by high-performance liquid chromatography coupled with mass spectrometry (HPLC-MS) using a Vanquish pump system and a TSQ Fortis triple-quadrupole as mass spectrometer (Thermo-Fisher Scientific, San Jose, CA, USA). The separation was achieved with a XBridge BEH C18 column (2.5 μm, 3.0 × 75 mm; Waters). The mobile phase consisted in water + 0.2% acetonitrile + 0.1% formic acid (eluent A) and acetonitrile + 0.1% formic acid (eluent B). Gradient elution was performed according to the following steps: isocratic 100% A for 1.8 min, from 100 A to 50% A by linear gradient in 11.4 min and 0.8 min at 50% A plus washing step at 0% A (100% B) and reconditioning. Flow rate was set at 0.2 ml/min, injection volume 5 μl and sample temperature 19°C. Detection was performed with a Waters SQ mass spectrometer with the following conditions: ESI source in positive ionization mode, source voltage 4.5 kV, source temperature 270 °C, desolvation temperature 200 °C. The acquisition was performed in SIM mode. As far as the analysis of tryptophan is concerned, 0.4 g of sample were hydrolyzed with 3 ml of 4 N NaOH at 100 °C for 4 h. Then the solution was neutralized, and the volume was brought to 100 ml with deionised water. The samples were then analyzed with HPLC instrument, consisting of two PU-1580 chromatographic pumps, an AS 1555 sampling system, a FP 1520 fluorescence detector (Jasco Corporation, Tokyo, Japan). The separation was performed with C8-Select B column; the mobile phase was Methanol:Water + 2% acetic acid (10:90); the flow rate was 1 mL/min. The fluorimeter was set at 280 nm excitation and 340 nm emission wavelengths. Quantifications were performed against a set of standard solutions.

### 2.3 In vitro protein digestibility and protein quality evaluation

*In vitro* protein digestibility has been determined with the method proposed by Minekus et al. (14) Accordingly, simulated saliva fluid (SSF), simulated gastric fluid (SGF), and simulated intestinal fluid (SIF) were created. For the oral phase, SSF containing human salivary α-amylase solution (1500 U/ml), CaCl_2_, and Milli-Q water were used. After adjusting the pH to 7, an incubation of 2 min at 37 °C on horizontal stirring (at 100 rpm) was run. The oral phase sample was combined with SGF containing a 25,000 U/ml pig pepsin solution and CaCl_2_ at 37 °C to mimic the stomach phase. After adding HCl to bring the mixture's pH down to 3, it was shaken at 100 rpm for 120 min at 37 °C. The stomach phase sample was then combined with bile salts and the SIF-containing pancreatin (800 U/ml) for the small intestine phase. Using NaOH, the mixture's pH was brought to 7.0. After that, the mixture was incubated for a further 120 min at 37 °C. After stabilizing with formic acid, all post-hydrolysis samples were centrifuged for 10 min at 23,000 g. The supernatants and solid residues were then kept at −20 °C until analysis. *In vitro* digestion of samples was done in triplicate.

The nutritional quality of proteins was determined by calculating the Protein Score and the DIAAS (Digestible Indispensable Amino Acid Score) on undigested bars. The Protein Score was calculated using the following formula (15):


$$\begin{array}{c} \text{P}\text{r}\text{o}\text{t}\text{e}\text{i}\text{n}\;\text{S}\text{c}\text{o}\text{r}\text{e}=\frac{\%\;of\;each\;essential\;amino\;acid\;on\;the\;\Sigma \;of\;essential\;amino\;acids\;in\;thebar}{\%\;of\;each\;essential\;amino\;acid\;on\;the\;\Sigma \;of\;essential\;amino\;acids\;in\;the\;referenceprotein}\times \;100 \end{array}$$ (1)

As reference protein was adopted the one proposed by WHO-FAO for subjects over 18 years of age and which corresponds only to the maintenance requirement, since beyond this age the WHO-FAO assume that there is no muscle growth (15).

The DIAAS was calculated using the in vitro digestibility coefficients of individual amino acids (digAA) to obtain the amount of digestible amino acid.


$$\begin{array}{c} \text{D}\text{I}\text{A}\text{A}\text{S}=\frac{\%\;of\;each\;essential\;amino\;acid\;on\;the\;\Sigma \;of\;essential\;amino\;acids\;in\;the\;bar{\;}^{*}digAA}{\%\;of\;each\;essential\;amino\;acid\;on\;the\;\Sigma \;of\;essential\;amino\;acids\;in\;the\;referenceprotein}\;\times 100 \end{array}$$ (2)

### 2.4 Digestibility of starch

Bars were washed by distilled water three times, after which they were soaked in distilled water with a weight ratio of 1:2 for 1 h at room temperature.

The *in vitro* starch digestion of bars was carried out following the Englyst method (16), based on the quantification of nutritionally relevant starch fractions, as rapidly digestible starch (RDS), slowly digestible starch (SDS) and resistant starch (RS). Samples were milled and then subjected to an acid-pepsin treatment to simulate gastric conditions. The intestinal phase was composed by an enzyme mixture containing pancreatin (P-7545, 8 × USP, Sigma, trypsin activity 6 U mg/1), amyloglucosidase (A 7095, Sigma) and invertase (I4504 Sigma). All enzyme solutions were freshly prepared. The RDS was the glucose release after incubation for 20 min, corrected for free glucose, the SDS as the glucose released between 20 and 120 min. Available starch (AS) was calculated as the sum of RDS and SDS. The SDS/AS ratio was calculated based on the SDS and AS contents. Samples were analyzed in triplicate. Resistant starch (RS) was measured using an enzymatic assay kit (Megazyme Ltd., Ireland).

### 2.5 Untargeted profiling of phenolic compounds

Phenolic compounds were extracted in triplicate (*n* = 3) from each raw corn flour following the method described by Rocchetti et al. (17), with some modifications. Briefly, 1 g of each corn sample was added with a hydro-alcoholic solution consisting of 80% methanol (v/v) (LC-MS grade, VWR, Milan, Italy) acidified with 1% formic acid. The extraction was promoted by using a Polytron™ PT1200E homogenizer (Kinematica, Malters, Switzerland), followed by centrifugation at 5,500 rpm for 15 min at 4 °C. Subsequently, the sample extracts were incubated overnight at −18 °C to promote protein precipitation. Finally, the different supernatants were filtered through 0.20 μm regenerated cellulose syringe filters and collected in amber vials until further instrumental analysis.

The corn samples were then analyzed by untargeted phenolic profiling, according to a high-resolution mass spectrometry

(HRMS) approach based on the utilization of a Q-Exactive™ Focus Hybrid Quadrupole-Orbitrap Mass Spectrometer (Thermo Scientific, Waltham, MA, USA) coupled to a Vanquish ultra-high-pressure liquid chromatograph (UHPLC), as previously reported in Rocchetti et al. (18). Briefly, for the full scan MS-data-dependent (Top *n* = 3) MS/MS mode, the positive ions were acquired in the range 80–1,200 m/z, exploiting a mass resolution of 70,000

FWHM in full scan and 17,500 FWHM in MS/MS mode, considering the three typical normalized collision energies used for fragmentation (i.e., 10, 20, and 40 eV). The chromatography and HESI parameters are described elsewhere (19). The generated raw data were then processed and statistically elaborated using the software MS-DIAL (version 4.90), reaching a level 2 of confidence in annotation through spectral matching against the comprehensive databases FooDB and Phenol-Explorer (mass accuracy < 5 ppm). The semi-quantification of the different phenolic classes was then carried out considering authentic standard compounds being selected as representative of their phenolic subclass (17). The results were finally expressed as mg Phenolic Equivalents (Eq.)/kg.

### 2.6 Statistical analysis

Data are expressed as mean ± standard deviation (SD) unless otherwise stated.

The effect of the type of corn used on starch digestibility has been analyzed by comparing results of the 3 different corn used for producing bars.

Statistical analysis was performed with SAS 9.4 software (SAS Inst., Cary, NC, USA) using the Bonferroni test. Statistical significance was set at *p* < 0.05.

As far as polyphenols are concerned, the one-way analysis of variance (ANOVA) was done using the software PASW Statistics 26.0 (SPSS Inc.) to identify significant differences (*p* < 0.05) in semi-quantitative values of each representative class of phenolic compounds in the different corn samples. Duncan's *post hoc* test was then used to identify homogenous subclasses (*p* < 0.05). Furthermore, the normalization of the untargeted dataset containing the different phenolics annotated was done using the software MetaboAnalyst 6.0. Raw data were normalized against the median values, log_10_ transformed and Pareto scaled before running the multivariate statistical analysis. This latter was based on the unsupervised hierarchical clustering (HCA; distance measure: Euclidean; Clustering method: Ward) that was created to group samples according to intrinsic similarities in their measurements (i.e., phenolic profiles).

## 3 Results

### 3.1 Chemical composition

Nutritional composition of analyzed bars are shown in Table 2. The products showed a high protein content (26.67%−38.14%), due to the presence of ham and meat as the main ingredient. Fat content ranged from 10.24 to 12.21% and carbohydrate (14.8%−18.4%), mainly consisting of starch.

**Table 2:** Chemical composition of the analyzed samples (g/100 g).

Sample	Energy^1^ (kcal)	Carbohydrates	Sugars	Protein	Fats	Fiber	Ash
RED-GEL5	306	18.0 ± 0.42	0.67 ± 0.04	32.66 ± 0.95	11.14 ± 0.23	3.02 ± 0.08	3.71 ± 0.27
BLU-GEL5	313	18.4 ± 0.28	0.58 + 0.03	34.39 ± 0.48	11.06 ± 0.24	2.59 ± 0.03	3.52 ± 0.19
AE-GEL5	287	14.8 ± 0.57	0.58 ± 0.05	32.28 ± 0.16	10.65 ± 0.14	5.53 ± 0.04	3.72 ± 0.31
AE-PSY5	300	17.4 + 0.60	0.67 ± 0.01	29.56 ± 0.20	12.21 ± 0.11	17.27 ± 0.89	4.12 ± 0.28
AEGEL10	307	14.9 + 0.14	0.67 ± 0.02	38.14 ± 0.20	10.24 ± 0.24	6.68 ± 0.32	3.58 ± 0.13
AE-PSY10	279	17.1 + 0.71	0.77 ± 0.05	26.67 + 0.22	11.18 + 0.01	30.76 + 0.64	4.16 ± 0.27

^1^calculated according to EU Regulation 1169/2011.

Products showed a medium energy content (279–313 kcal/100 g).

Dietary fiber ranges from 2.59 to 30.76%. GEL 5 (excepted for BLU-GEL 5) presented a significant amount of fiber, higher than 3g/100 g (which can be claimed “source of fiber”), while GEL10 were all “rich in fiber” (10). As expected, the samples with PSY 5 and PSY 10 were characterized by a higher fiber content (17.27 g and 30.76 g, respectively) than the other samples.

### 3.2 Untargeted phenolic profile of corn samples

The untargeted phenolic profiling based on UHPLC-HRMS allowed the putative annotation of 490 compounds, including: 291 flavonoids (comprising different subclasses, such as flavan-3-ols, anthocyanins, flavonols, flavones, and flavanones), 100 phenolic acids (including both hydroxycinnamics and hydroxybenzoics), 11 stilbenes, and 91 other remaining phenolics. All the identified compounds are reported in supplementary material, organized in their corresponding phenolic class, and considering their monoisotopic mass spectra and additional mass spectrometry-based information (such as adduct type and total identification score).

The results of the semi-quantitative analysis on the different phenolic subclasses are then summarized in Table 3. Overall, significant differences (*p* < 0.05) were outlined by considering the distribution of anthocyanins, other flavonoids (i.e., flavone equivalents), other phenolics, phenolic acids, and stilbenes. No significant differences (*p* > 0.05) were found for both flavan-3-ols and flavonols in the different corn samples. The highest total phenolic content (evaluated as cumulative value of the different phenolic subclasses) was recorded for yellow corn (1166.75 mg/kg), followed by blue corn (866.17 mg/kg) and red corn (717.16 mg/kg). Anthocyanins were significantly higher in blue corn, followed by red corn and yellow corn; in this regard, blue corn and red corn were particularly abundant in delphinidin 3-*O*-6”-acetyl-galactoside), and different cyanidin glycosides (such as succinyl-glucosides and sambubiosides; supplementary material). Blue corn was also the most abundant source of luteolin equivalents (Table 3). As far as the distribution of phenolic acids is concerned, yellow corn showed a high concentration for this phenolic class, recording high abundance values for feruloyl- and diferuloyl- putrescine, followed by protocatechuic acid and gallic acid (supplementary material). Among the identified phenolic compounds, we found no significant differences (*p* > 0.05) in the distribution of ferulic acid, caffeic acid, and coumaric acid, although *ad-hoc* works should be carried out considering that these compounds mainly exist in this matrix as bound phenolic fraction. Yellow corn was characterized by high contents of other phenolics, being particularly abundant in carnosol, a naturally occurring phenolic diterpene. Other abundant compounds belonging to this phenolic subclass were catechol and 4-hydroxybenzaldehyde, showing similar abundance values in the test corn flours (supplementary material). Finally, stilbenes was the phenolic class consisting in the lowest number of annotated compounds; yellow corn was again the richest source, followed by red corn and blue corn (Table 3). Overall, resveratrol 5-*O*-glucoside was a phenolic compound showing a similar distribution in all the tested samples (supplementary material).

**Table 3:** Semi-quantitative analysis of the different phenolic classes annotated in the different corn samples (mg of phenolic equivalents/kg).

Phenolic class equivalents (EQ)	Blu corn	Red corn	AE yellow corn
Anthocyanins (Cyanidin 3-rutinoside Eq.)	218.56 ± 13.38^c^	166.32 ± 16.89^b^	12.35 ± 0.53^a^
Flavan-3-ols (Catechin Eq.)	8.29 ± 1.50	9.31 ± 1.47	10.63 ± 1.33
Flavonols (Quercetin Eq.)	5.90 ± 1.13	4.76 ± 1.12	5.33 ± 1.94
Other flavonoids (Luteolin Eq.)	163.87 ± 1.54^c^	112.01 ± 1.96^a^	141.97 ± 5.22^b^
Other phenolics (Oleuropein Eq.)	207.59 ± 9.45^b^	172.23 ± 1.26^a^	422.47 ± 0.86^c^
Phenolic acids (Ferulic acid Eq.)	265.20 ± 12.45^a^	248.69 ± 10.79^a^	568.64 ± 10.19^b^
Stilbenes (Resveratrol Eq.)	3.42 ± 0.43^a^	3.85 ± 0.28^a^	5.37 ± 0.29^b^
Total phenolics	866.17	717.16	1166.75

Results are expressed as mean value ± SD (*n* = 3). The different superscript letters within each row indicate significant differences as resulting from ANOVA and Duncan *post hoc* analysis (*p* < 0.05).

**Figure 1:**
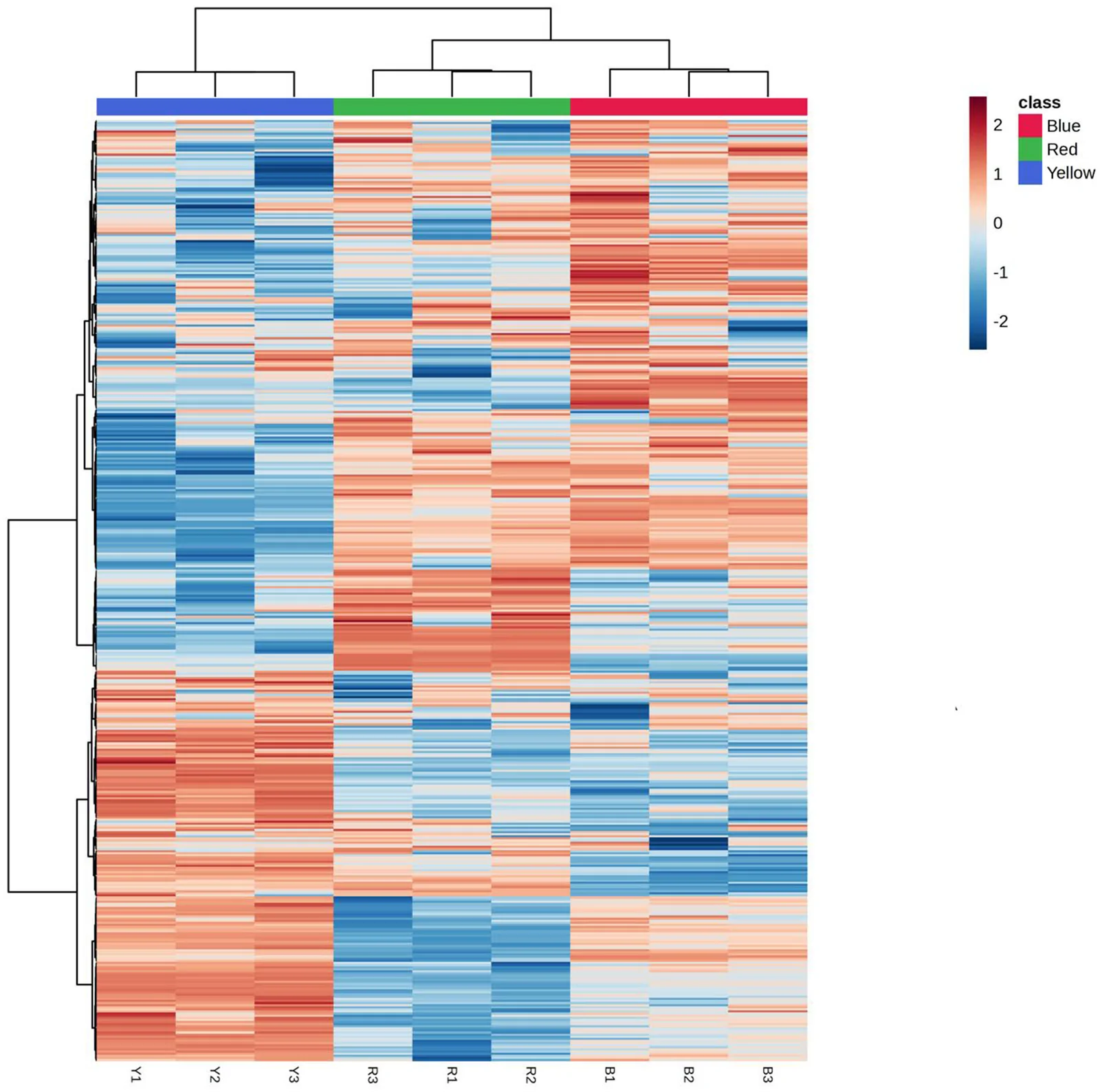
Heat map resulting from unsupervised hierarchical cluster analysis carried out considering the phenolic profile of the different corn samples.

Regarding multivariate data analysis, the unsupervised heat map reported in Figure 1 (based on the fold-change distribution of each annotated phenolic compound) was able to group corn samples according to the similarities in the phytochemical profiles. Accordingly, the heat map showed a good discrimination as a function of pigmentation. Accordingly, blue and red corn samples clustered together, while yellow corn was included in an exclusive cluster (Figure 1). The red and blue color within the heat map is indicative of up- or down-accumulation of phenolics, respectively.

### 3.3 Protein digestibility and quality

The *in vitro* protein digestibility of all analyzed samples (Table 4) ranged from 95.3 to 96.2. The high digestibility of the bars is likely due to the inclusion of raw ham in the bar, whose proteins have been partially hydrolyzed during curing (at least 10 months).

**Table 4:** Protein and amino acid digestibility and protein score (DIAAS, Digestible Indispensable Amino Acid Score) of the samples.

Amino acid	Type of snack bars					
	RED-GEL 5	BLU-GEL 5	AE-GEL 5	AE-PSY 5	AE-GEL 10	AE-PSY 10
Protein digestibility (%)	95.5	95.9	95.5	96.2	95.3	96.2
Amino acids digestibility (%)						
Thr	96.24	96.60	96.49	96.31	96.01	95.55
Val	95.78	96.21	95.71	96.39	96.09	96.51
Ile	95.96	96.52	95.81	96.54	96.71	96.79
Leu	96.26	96.76	96.22	96.71	96.73	96.97
Tyr	96.83	97.21	96.54	96.99	97.45	97.09
Phe	96.05	96.60	96.24	96.67	96.38	97.02
His	96.76	96.35	95.96	96.12	95.91	96.58
Lys	95.48	95.98	95.45	96.00	95.64	96.45
Trp	97.66	97.91	98.20	97.38	98.09	97.35
Cys	97.77	97.19	97.98	97.42	97.75	97.62
Met	96.18	94.57	97.10	96.39	96.62	96.94
DIAAS	74.3	77.3	74.4	74.6	79.6	75.1
1^st^ limiting amino acid	Val	SAA	Val	Val	Val	Val
2^nd^ limiting aminoacid	Leu	Val	Leu	Leu	Leu	Leu

The essential amino acid content and protein score are shown in Table 5. With the exception of BLU-GEL 5, where sulfur amino acids are the limiting ones, in all the other products examined, the limiting amino acid is valine. The PS values are good and range from 75 to 80.

**Table 5:** Essential amino acid (EAA) content (g of single EAA/Σ EAA) and Protein Score of the six snack-bars.

Amino acid	Type of snack bars						Amino acid maintenance requirement (expressed as single EAA/Σ EAA) according to WHO-FAO^1^
Amino acid	Sample						
	RED-GEL 5	BLU-GEL 5	AE-GEL 5	AE-PSY 5	AE-GEL 10	AE-PSY 10	
Thr	10.28	10.67	11.16	9.21	9.95	9.33	8.3
Val	10.50	11.02	10.53	10.51	11.25	10.60	14.08
Ile	9.17	10.14	9.00	10,08	9.73	10.29	10.83
Leu	17.02	18.14	16.52	17.45	17.61	17.48	21.3
Tyr	6.62	7.28	6.21	7.02	6.84	7.06	
Phe	8.56	9.03	8.42	8.38	9.20	8.56	
Aromatic amino acids (AAA)	15.18	16.31	14.63	15.40	16.04	15.62	13.72
His	9.88	8.89	8.46	9.00	8.13	8.83	5.42
Lys	14.92	16.29	15.64	15.81	16.23	16.12	16.25
Trp	3.00	2.35	2.92	2.68	2.12	2.63	2.17
Cys	3.16	2.44	3.41	3.22	2.68	3.06	
Met	6.89	3.75	7.74	6.63	6.27	6.05	
Sulfur amino acids (SAA)	10.04	6.19	11.15	9.85	8.94	9.11	7.94
Protein score	74.6	78.0	74.8	74.7	79.9	75.3	
1^st^ limiting amino acid	Val	SAA	Val	Val	Val	Val	
2^nd^ limiting aminoacid	Leu	Val	Leu	Leu	Leu	Leu	

^1^Millward (15).

If we consider the DIAAS (Digestible Indispensable Amino Acid Score) the limiting amino acids remain the same as the Protein Score and also the DIAAS values are practically superimposable to the Protein Score calculated on the undigested product (Table 4). The ratio of branched chain amino acids (Leu: Ile: Val) indicates a lower concentration of Ile (0.86–0.97) and especially of Leu (1.57–1.66), compared to Val.

### 3.4 Starch digestibility

**Figure 2:**
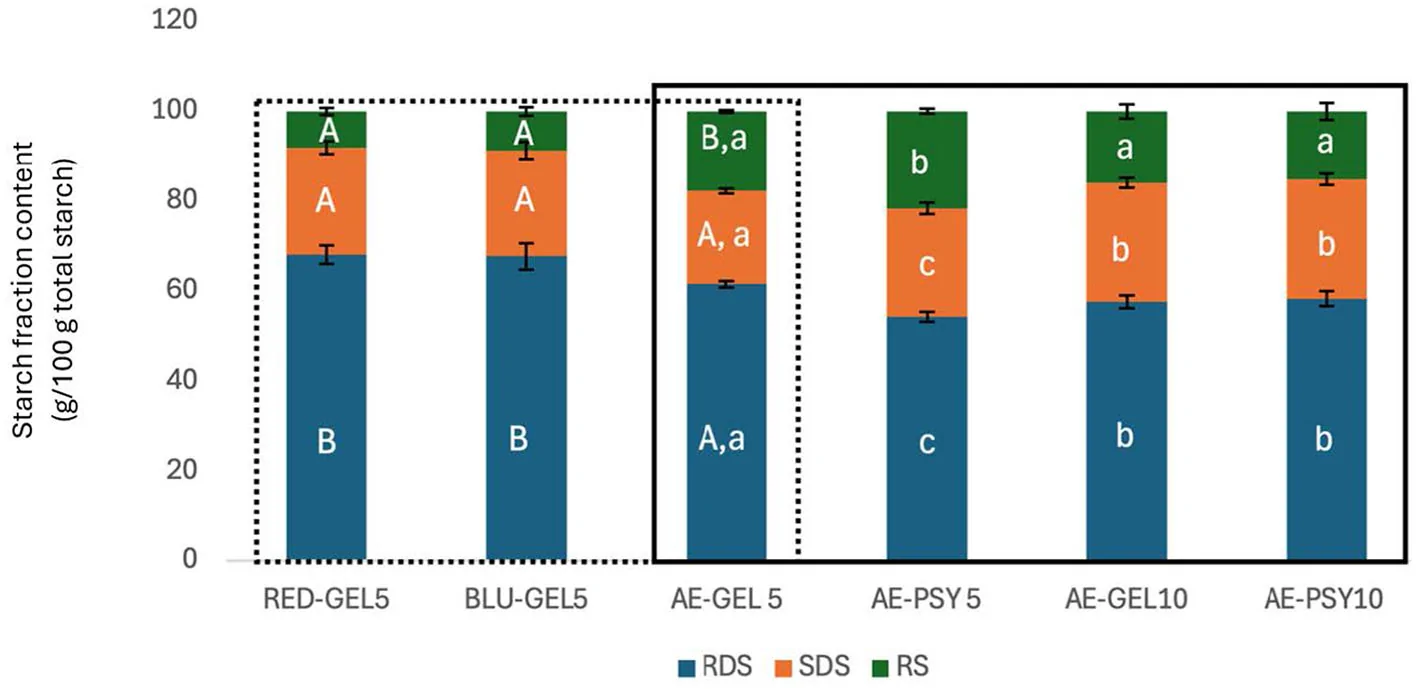
Starch fraction content (g/100 g total starch) considering rapidly digestible starch (RDS), slowly digestible starch (SDS) and resistant starch (RS) in experimental samples. RED-GEL5: red pigmented corn with 5% pork gel; BLU-GEL5: blue pigmented corn with 5% pork gel; AE-GEL5: yellow high amylose corn with 5% pork gel; AE-PSY5: yellow high amylose corn with 5% psyllium fiber; AE-GEL10: yellow high amylose corn with 10% pork gel; AE-PSY10: yellow high amylose corn with 10% psyllium fiber. Dotted lined square: A, B: *P* < 0.05, effect of the type of corn within the same level of pork gel in the recipe. Continuous lined square: a, b, c: *P* < 0.05, effect of the level of two different gelatinizing agents (i.e., 5 and 10 % in the recipe of pork gel or psyllium fiber) within the same type of corn (i.e., psyllium fiber). *P*-value of the contrast within the effect of the level of two different gelatinizing agents (i.e., 5 and 10 % in the recipe of pork gel or psyllium fiber) within the same type of corn (i.e., psyllium fiber): AE-GEL5 vs. AE-PSY5: RDS *P* < 0.05; SDS *P* < 0.05; RS *P* < 0.05; AE-GEL10 vs. AE-PSY10: RDS ns; SDS ns; RS ns; AE-PSY5 vs. AE-PSY10: RDS *P* < 0.05; SDS *P* < 0.05; RS *P* < 0.05; AE-GEL5 vs. AE-GEL10: RDS *P* < 0.05; SDS *P* < 0.05; RS ns.

AE-GEL 5 has a lower rapidly digestible starch fraction (RDS) than pigmented corn (Figure 2). No significant difference was observed for the slowly digestible fraction (SDS). Resistant starch was significantly higher in AE-GEL 5 than in RED-GEL 5 and BLU-GEL 5 (Figure 2). The comparison between the different types of gelling agent and their dosages is shown in Figure 2. The RDS fraction increased with the use of GEL compared to PSY, while increasing the level of inclusion of the gelling agent from 5 to 10% determined an opposite result, with GEL reducing the RDS fraction as its inclusion in the dough increased, while PSY caused the opposite effect.

The two different gelling agents also significantly influence the SDS, but with an opposite trend compared to the RDS fraction. In this case, it is the PSY that increases the percentage of this fraction: from 24.1 to 26.5%. The increase in the percentage of inclusion of the gelling agent determines an increase in the SDS with both ingredients.

Resistant starch decreases with increasing gelling agent inclusion level, but while no dose effect was observed for GEL, in bars produced using PSY, the RS fraction decreases from 21.6 to 15.2% when the PSY inclusion level increases from 5 to 10%

Starch digestibility was lower in products where the binding agent was Psyllium fiber instead of pork gelatine.

## 4 Discussion

The reuse of by-products from the pork meat industry and their combination with unconventional starch sources and gelling fibers, used as product binders, enables the production of innovative foods and greater environmental sustainability of this production chain.

Since the ingredients were weighed as is and not dried, the quantity of psyllium used, if expressed on the dry substance, is greater than that calculated with ingredients containing water.

The fact that the addition of this gelling agent, as well as of Psyllium fiber, determines fiber contents higher than those theoretically expected on the basis of the recipe, could be explained by the fact that the ingredients were weighed as is and not dried, therefore the quantity of psyllium used, if expressed on the dry substance, is greater than that calculated with ingredients containing water. Baking, by modifying the water content of the bars, also introduces a further variation factor compared to the theoretical calculation. The reason for this discrepancy, however, is likely due to the fact that the method used to determine fiber (AOAC 2017.16) allows for the determination of several oligosaccharides, such as arabinoxilans (which constitute the backbone of corn hemicellulose) as well as resistant starch (20), which is abundant.in the corn utilized, mainly in the amylose extender variety. Indeed, the dietary fiber contents determined using the AOAC 2017.16 method were 43.47% for “amylose-extender” maize, 24.69% for blue maize, and 25.31% for red maize. A further hypothesis that could explain the high fiber levels in the bars produced with psyllium is a reduction in the accessibility of the starch to alpha-amylase enzymatic treatment; additionally, thanks to its gelling capacity, psyllium fiber can entrap proteins, preventing them from precipitating and subsequently being removed during the ethanol treatment prescribed by the 2017.16 method. A fiber content higher than expected (approximately 50% higher) had previously been observed in biscuits made with wheat flour and psyllium inclusion levels ranging from 3 to 9% (21). As the AOAC 2017.16 method was not yet available, those authors had used the AOAC 991.43 method, suitably modified to adapt it to products containing psyllium fiber (22).

A product like the bars developed in this trial, could be useful in two situations that require an increased intake of protein and BCAAs.

The first is sarcopenia, a muscle disease that results in the progressive loss of muscle mass and strength and is predominantly associated with old age. A review by Papadopoulou (23) highlights the presence of sarcopenia in between 5 and 33% of elderly people living at home, 15%−35% in those hospitalized and 38%−40% in those admitted to nursing homes.

In fact, this condition is related to both an inflammatory process, which can be prevented by dietary factors such as fruits, vegetables, whole grains, and essential fatty acids from fish products, and a protein deficiency, which is why increased intake of branched chain amino acids (BCAAs) is shown to be effective in preventing and treating sarcopenia.

Another nonpathological condition in which increased protein intake plays a relevant role is sports. In sports nutrition, there is a need to increase both the amount of protein ingested (from 0.8 to 1 g/kg of body weight to 1.4–1.7 g/kg of body weight) and the quality, particularly branched chain amino acids, which can increase protein synthesis after intense physical activity, thus facilitating the athlete's recovery (6).

Although the Protein Score and DIAAS indicate valine as the predominantly limiting amino acid, the analysis of the ratios between the concentrations of branched-chain amino acids indicates a relative deficiency of leucine, whose concentration compared to valine is decidedly lower (1.57–1.66) than the value considered adequate (2.0) (15).

Since this amino acid is particularly important in determining muscle protein synthesis (24), its deficiency compared to valine indicates the need to increase its concentration in products.

The high DIAAS values obtained in this study were achieved *in vitro*, where conditions are optimal; therefore, it is not guaranteed that such high digestibility values would also be obtained *in vivo*

In particular, the gelling capacity of psyllium fiber can hinder the access of digestive enzymes to the substrate in the gastrointestinal tract, resulting in lower digestibility compared to *in vitro* values.

Furthermore, it should be noted that industrial-scale production of these bars—in order to ensure a long shelf life—may require heat treatments more intense than those used in this prototype production (110 °C for 2.5 h). The use of higher temperatures reduces lysine availability due to the Maillard reaction.

The lower susceptibility to amylase attack by high-amylose starch is the likely explanation for the lower pGI in bars made with yellow corn compared to those made using red or blue corn.

When psyllium fiber supplementation is increased from 5 to 10%, rapidly digestible starch (RDS) decreases, likely because the fiber absorbs water and limits amylase access to the substrate.

For this very reason, the reduction in the RDS percentage results in an increase in the proportion of slowly digestible starch (SDS).

From a regulatory and functional perspective, the compositional characteristics of the developed bars support several nutrition and health claims. All formulations provide more than 20% of their total energy from protein, thereby meeting the requirements to bear the claim “high protein” (≥20% of energy from protein) in accordance with Regulation (EC) No 1924/2006. (19). This represents a significant added value in light of the growing consumer demand for high-protein foods.

However, it should be noted that, according to ESPEN guidelines (25), the prevention and treatment of sarcopenia rely not only on adequate amino acid intake from a single food source but also require sufficient energy intake and the inclusion of plant-based anti-inflammatory compounds. Physical activity also plays a crucial role in maintaining bone and muscle function. Therefore, the bars developed in this study are suitable for inclusion in diets addressing increased protein requirements, although their clinical efficacy has yet to be demonstrated.

The addition of 10% psyllium fiber enables certain formulations to claim products “high in fiber” (≥6 g/100 g), further enhancing their nutritional profile. Although pork gelatine present in the formulation could theoretically support a “source of fiber” claim, this should be verified, as it may be classified as dietary fiber from an analytical perspective (20), despite not being of plant origin.

The resistant starch (RS) content of bars produced with yellow corn AE accounts for 17.8% of total starch and therefore possibly claimed “reduction of post-prandial glycaemic response”, provided that resistant starch represents at least 14% of total starch, in accordance with Commission Regulation (EU) No 432/2012. Several authors have developed snack products starting from by-products of the food industry exclusively of plant-based origin, both widely cultivated: pear and apple (26) bean flour (27) but also with a more limited geographical diffusion: jabuticaba peel and okara (28) and cerola residues (30).

Ribeiro et al. (29) developed 6 different types of snack bars mixing by-products of maize flour processing, having high fiber content, with cereal flakes (rice, oat). These new foods, unlike the high-protein snack bars developed in this research, are characterized by low protein contents: from 4.2 to 6.1% in the work of Bchir et al. (26) and 7% for the bar with the best sensory properties in the work of Ribeiro et al. (29). On the other hand, the carbohydrate intake is very high, being equal to 55.4% in the work of Ribeiro et al. (29) while for the products developed by Bchir et al. (26) this parameter oscillated between 55.5 and 60.4%. The carbohydrates included in these bars are mainly simple sugars, while the carbohydrate content of the similar products developed in our research, in addition to being much lower (> 20%), is mainly made up of corn starch, which is digested more slowly than simple sugars.

## 5 Conclusions

The valorization of the residues from the slicing of raw ham, combined with a starch source with an interesting nutritional profile such as unconventional corn cultivars, allows the creation of protein bars useful for situations that require a higher protein intake.

Compared with pigmented corn (red and blue), yellow corn with a high amylose content showed a higher share of resistant starch, while digestibility showed no differences with other varieties of corn. Psyllium fiber increases the fraction of resistant starch compared to porcine gelatine and increasing its inclusion level from 5 to 10% accentuates this reduction. No dose effect was observed in porcine gelatine. The Protein Score values are quite high and are between 75 and 80, with valine as the main limiting amino acid. The situation is substantially identical for the PDCAAS, both for the values of this index and the limiting amino acids, identical to those highlighted with the Protein Score. All six formulated products can carry the claim “Rich in protein” on the label, while the claim rich in fiber can only be attributed to the bars added with Psyllium fiber. Yellow corn with gelatin can be considered a “source of fiber” according to EU Regulation (18). This study demonstrates how residues from the meat industry can be adequately valorized for the production of high protein products, thus reducing the by-products of the meat industry.

Our work is preliminary and has several limitations, including the absence of “*in vivo*” testing; consequently, the actual effects on muscle protein synthesis, glycemic response, and metabolism in general remain unknown and will require future investigation.

Furthermore, the organoleptic properties of the six bars produced were not evaluated.

## Data Availability

The raw data supporting the conclusions of this article will be made available by the authors, without undue reservation.
